# Numerical simulation of the influence of building-tree arrangements on wind velocity and PM_2.5_ dispersion in urban communities

**DOI:** 10.1038/s41598-022-20455-6

**Published:** 2022-09-30

**Authors:** Fan Li, Matteo Rubinato, Tao Zhou, Jiaye Li, Chen Chen

**Affiliations:** 1 School of Environment and Civil Engineering, Dongguan University of Technology, Dongguan, 523808 China; 2 Key Laboratory of Engineering Software, Center for Hydrosphere Science, Dongguan University of Technology, Dongguan, 523808 China; 3 Centre for Agroecology, Water and Resilience, Coventry University, Coventry, CV8 3LG UK; 4 School of Management Science and Real Estate, Chongqing University, Chongqing, 400044 China

**Keywords:** Environmental impact, Civil engineering

## Abstract

Airflow behavior and outdoor PM_2.5_ dispersion depend significantly on the building-tree layouts and orientation towards the prevailing wind conditions. To investigate this issue, the present work evaluates the aerodynamic effect of different building-tree layouts on the outdoor PM_2.5_ dispersions in the urban communities of Shijiazhuang City, China. The adopted numerical CFD technique was based on the standard k–ε model and the Disperse Phase Model (DPM). For this study, ten different building-tree arrangements were conceptualized and all these configurations were simulated by using Ansys Fluent software to quantify the implications on the outdoor PM_2.5_ dispersion due to their presence. The results have shown that: (1) a wide building interval space could benefit the air ventilation and thus decrease PM_2.5_ concentrations, however, this effectiveness is highly influenced by the presence of the trees; (2) the trees on the leeward side of a building tend to increase the local wind velocity and decrease the pedestrian-level PM_2.5_ concentrations, while those on the windward side tend to decrease the wind velocity. The small distance with trees in the central space of the community forms a wind shelter, hindering the particle dispersion; and (3) the configuration of parallel type buildings with clustered tree layouts in the narrow central space is most unfavorable to the air ventilation, leading to larger areas affected by excessive PM_2.5_ concentration.

## Introduction

With the rapid urbanization and industrialization, China has been experiencing serious environmental pollutions, especially the air pollution^1^. According to the Environmental Performance Index, China ranked worst out of 175 countries in the world for air quality^2^ in 2018. In recent years, many cities across the country have experienced serious haze effect caused by atmospheric suspended particulate matter, and this phenomenon is becoming increasingly more frequent^3^. The pollution of fine particulate matters not only affects the people’s normal life, but also poses a great threat to their physical and mental health^4^. For people living in large cities, the green areas and residential communities are two of the most important places to stay and conduct recreational activities, and it was estimated that people spend more than 40% of their time within these locations^5^. Thus, the fact that the residents are unfortunately directly exposed to the pollutants of particulate matter in the air created a strong concern, and this has become an urgent issue that the municipalities should take into consideration.

The diffusion trajectories of gaseous pollutants mixed within the air essentially depended on the direction of the wind^6^, especially for some fine-size particulate matters^7^. The built environment of the residential districts, such as the layout of buildings and greening design, can affect the velocity and the direction of airflows, thus affecting pollutant dispersion in urban areas^8^. To date, the methods used to explore airflow and pollutant diffusion in urban environments mainly include the field measurement, wind tunnel test, and Computational Fluid Dynamics (CFD) simulation^8^. Hong et al.^8^ adopted a numerical simulation using Reynolds-Averaged Navier–Stokes (RANS) model to investigate the coupled effects of different building-tree arrangements on outdoor PM_2.5_ dispersion in housing blocks and provided results that can support future sustainable projects to maximize particle removal capability. However, given the rapid development of technology and the continuous improvement within numerical computing techniques, CFD has been considered as a promising tool for parametric studies of airflow and dispersion process at micro scale^9^. In fact, Gousseau et al.^9^ conducted high resolution CFD simulations for neutral atmospheric conditions and validates experimental results obtained via a wind-tunnel. This was one of the initial CFD studies of urban dispersion which focused on the far-field spread of contaminants and obtained high-resolution results.

Several studies have implemented CFD technology to investigate the influence of different forms and layouts of buildings on the dispersion of air pollutants at different scales^10–14^. These studies found that both the building characteristics and the layout can influence the air ventilation and, thus, the diffusion of air pollutants^11,12^. Several community-scale numerical studies have compared the airflow characteristics under different configurations of building arrangement. For example, Ma et al.^13^ studied six basic building layout forms based on the arrangement characteristics of forty-one residential districts in Xi’an, China and found that the u-shaped building arrays were relatively most conducive to air ventilation, thus promoted PM_2.5_ dispersion. Tao and Li^14^ comparatively analyzed the natural ventilation of three typical residential building arrangements in Hefei, China, and found that the staggered- and enclose-type buildings have the best and worst wind environment, respectively.

In addition to the building, plants are another important component in the residential districts that are believed to influence the airflow and particle dispersion^8^. Jeanjean et al.^15^ found that PM_2.5_ concentrations were reduced by 9.0% due to the aerodynamic dispersive influence of trees during the Summer in Leicester, UK. It has been found that a tall and dense leaf vegetation can generate turbulent air movements and thus capture significant amounts of the particulate matter^15,16^. It was also found that each different tree species can capture dissimilar quantities of particles^17^, and the trees with small stems and large leaves could increase the flow velocity with which the particles would deposit^18^. Additionally, several other numerical studies have identified that the height, species, leaf density and crown size of the trees may also impact the airflow and particle dispersion^19,20^.

Despite lots of work having been conducted to study the effect of various factors on the air pollution, very few have considered the combined effects of building and tree on the diffusion of pollutants. For example, Hong et al.^8^ explored the aerodynamic effects of nine building-tree configurations on PM_2.5_ dispersion. It was found that the buildings, trees, and their relative positions all have an impact on the spatial distribution of the pollutants. However, the building-tree configurations used in this study were mainly obtained from a very small urban residential area.

There are also some other studies that explored the influence of buildings or trees (and even their combined effects) on PM_2.5_ dispersion at different scales, such as city-, block- and neighborhood-ones. To the author’s knowledge, almost no studies have reported on the coupled effects of building-tree arrangement on PM_2.5_ dispersion at the community scale, and this is a promising field that requires further investigation because the geometry and location appears to be crucial for the characterization of the wind fields and consequently for the estimation of the concentration of pollutants. By identifying these features, it could be possible to achieve an optimal configuration or provide an evaluation index for architectural and tree planting design process. Buildings and trees are vital within any community and considering that they both independently have an impact on the air pollution and the spread of particles (affecting the wind conditions) within the air, it would be crucial to identify which layout could generate a minimal negative impact on the environment. Furthermore, by exploring the aerodynamic effects of building-tree arrangement on PM_2.5_ dispersion, we can also have more understanding regarding the mechanism for reducing PM_2.5_ exposure. The results will lead to the optimization of the arrangement of buildings and trees, targeting a more sustainable design for the urban communities.

## Methodology

### Study area and sampling procedure

**Figure 1:**
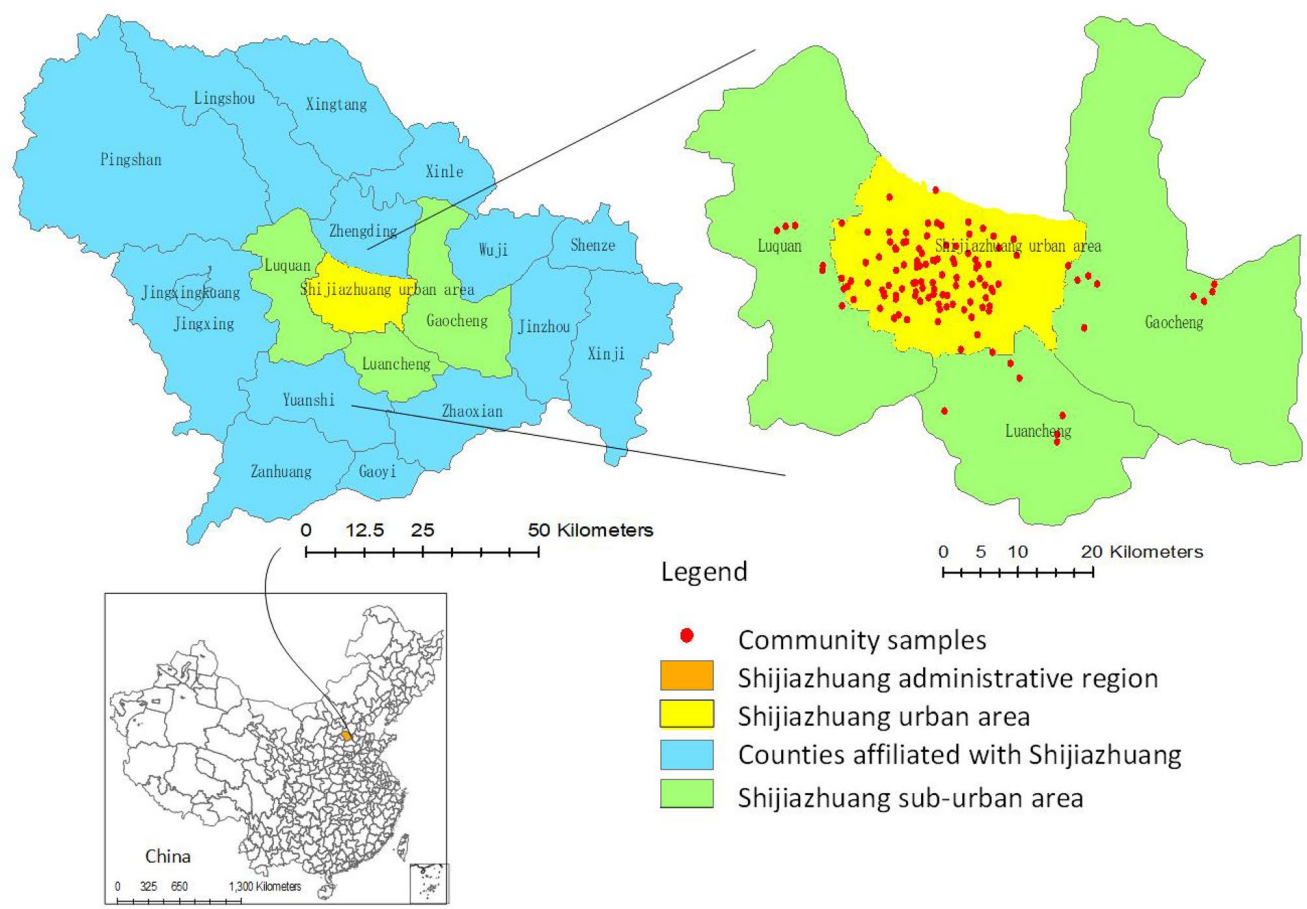
Study area and community sample distributions (made with acgGIS10.2, https://support.esri.com/en/download/2089).

Shijiazhuang City is the capital of Hebei province, located in the Northern China (37° 27′–38° 47′ N, 113° 30′–115° 20′ E) (Fig. 1). It is an ancient industrial city including textile, pharmaceutical, steel and chemical productions. These have caused severe air pollutions over the last decades and therefore, Shijiazhuang has been continuously ranked as one of the most polluted cities in China^21^. According to the data released by the *China National Environmental Monitoring Center*, from 2013 to 2017, excessive PM_2.5_ and PM_10_ concentration days were much longer than those of the other pollutants in Shijiazhuang, especially in the Winter season. This makes it essential to investigate the aerodynamic influence of the built environment on PM dispersions in selected residential areas of Shijiazhuang for the purpose of reducing the people’s exposure to health risks.

To gather all necessary information of the building-tree arrangement within the communities living in the urban areas of Shijiazhuang, a commercial housing platform called Anjuke was used, which provides comprehensive housing and community details. During the sample selection, a total of 146 blocks were identified. Given the repeatability of some similar configurations within the dataset, a representative layout for each category was identified. Meanwhile, two other specific criteria were also considered: firstly, the construction completion date for all the selected communities should be post-2005 in order to reflect the prevailing design preference; secondly, the selected communities should include more than three building blocks with detailed records on the building-tree arrangement. Considering these guidelines, a total of 120 communities have been identified and their locations are also shown in Fig. 1.

The building-tree arrangements in these 120 communities have the following features: The layouts of the buildings mainly include the parallel type, the staggered type, the semi-enclosed and the enclosure types. Moreover, the trees are mainly located around the buildings and within their central space. The statistical characteristics of the arrangement of buildings and trees are shown in Table 1.

**Table 1:** Statistics of the arrangement of buildings and trees in selected communities of Shijiazhuang City.

		Building layouts					
		Parallel type	Semi-enclosed type	Enclosure type	Staggered type	Other type	Total
Tree layouts	Around buildings	18	0	0	1	3	22
	Central spaces	5	4	4	2	2	17
	Around buildings and central spaces	40	19	14	7	1	81
	Total	63	23	18	10	6	120

**Figure 2:**
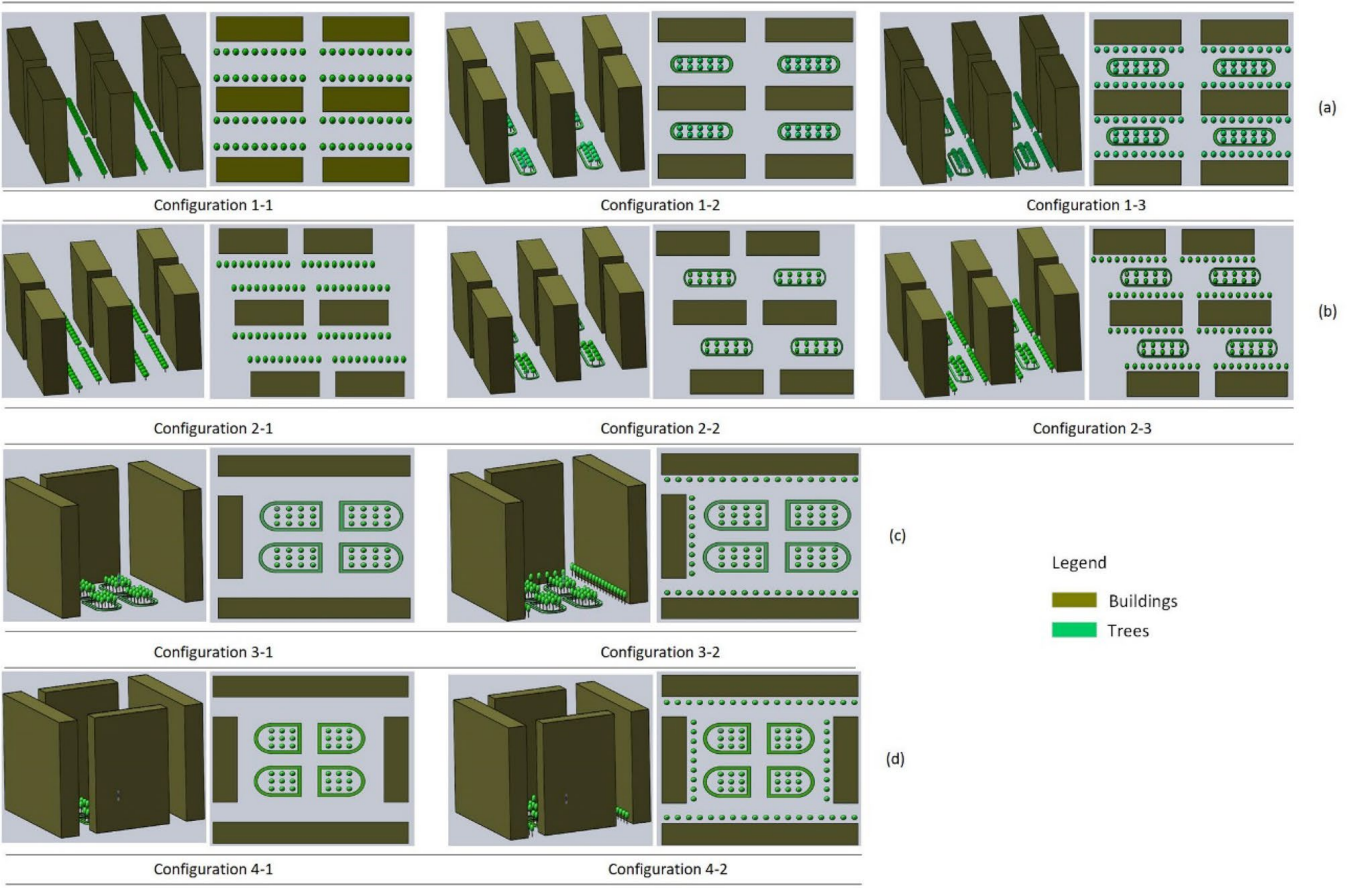
Various patterns of building-tree arrangements in the study: (**a**) parallel type; (**b**) staggered type; (**c**) semi-enclosed type; and (**d**) enclosure type.

According to Table 1, there are totally 10 typical building-tree arrangements, as schematically shown in Fig. 2. The “other type” building layouts were excluded due to their low representation in the samples, i.e. only 6 out of 120 samples.

According to the *Urban Residential Greenbelt Layout Design Standard in Hebei Province* (DB13/T1347-2010), the greening design of the residential areas should adopt a variety of plant forms, such as trees, shrubs and lawns. In present study, given the fact that the grasses are very short and thus have very limited aerodynamic effects on the airflow, they are excluded from the consideration. Among the 120 samples in Table 1, the greening form around the building is mainly represented by trees, which accounts for 69.2% of the total sample. The combination of trees and shrubs is the most common greening form inside the building interval, which accounts for 76.7% of the total sample. This greening combination with the largest proportion in the 120 samples was used when constructing the building-tree arrangement models. It should be noted that the current study mainly focuses on the influence of relative position between the buildings and trees on the airflow. Therefore, the individual difference of tree and shrubs forms, such as their specific size, height etc. in each model was not considered.

### Computational approach

The numerical simulation is based on Ansys Fluent software and the standard *k*-*ε* model is used for the airflow simulation because it is a common modeling technique for various problems related to the air flow and pollutant transport^22^. Many studies have used the *k*–*ε* model to simulate the aerodynamic influence of buildings or trees on pollutant diffusion^8,10,23^; they compared the results of numerical simulation and field measurement and found good agreements between them, confirming the reliability of the *k*–*ε* model to accurately simulate turbulence. However, the standard *k*–*ε* model may overestimate turbulent kinetic energy, leading to more discrepancies and a less accurate simulation of vertical turbulence near the building edge area^24^. This study mainly focuses on the horizontal integrated turbulence of 1.5 m pedestrian height, where the vertical turbulence near the buildings is relatively small, therefore, this enabled the use the standard *k*–*ε* model producing reliable and accurate results. The main aerodynamic effect of buildings and trees on the wind field is to change the velocity and direction of the air through their friction and form drag forces. As such, the turbulent effect of different arrangements on the air flow should be quantified.

PM_2.5_ is a kind of solid particulate pollutant. There are two common numerical methods to track the motion of suspended particles within the air, which are the mixture model and Disperse Phase Model (DPM). The former is applicable when the volume fraction of the discrete phase is large, and the medium can be treated as a continuum. The latter is mostly used in the case that the volume fraction of the discrete phase is small inside the flow field, when the particle trajectory of a certain phase needs to be tracked. This study adopted the DPM method to simulate the interphase coupling of PM_2.5_ and the air flow, for the purpose of tracking the motion of PM_2.5_ pollutant diffusion.

### On-site measurement

For this study a community in Yuhua District, within Shijiazhuang city, was used to carry out the field measurement of wind velocity and PM_2.5_ concentration for the purpose of calibrating the numerical model parameters. This community was chosen because the buildings in this area are laid out in a parallel style, and the greenery is located around the buildings, therefore constituting a layout that is most representative of the building-tree arrangement of communities in the same area. In addition, the surrounding environment is relatively empty and lacks the aerodynamic influence of other tall buildings, which provide simple boundary conditions to model the airflow and diffusion of pollutants within the ambient flow field.

**Figure 3:**
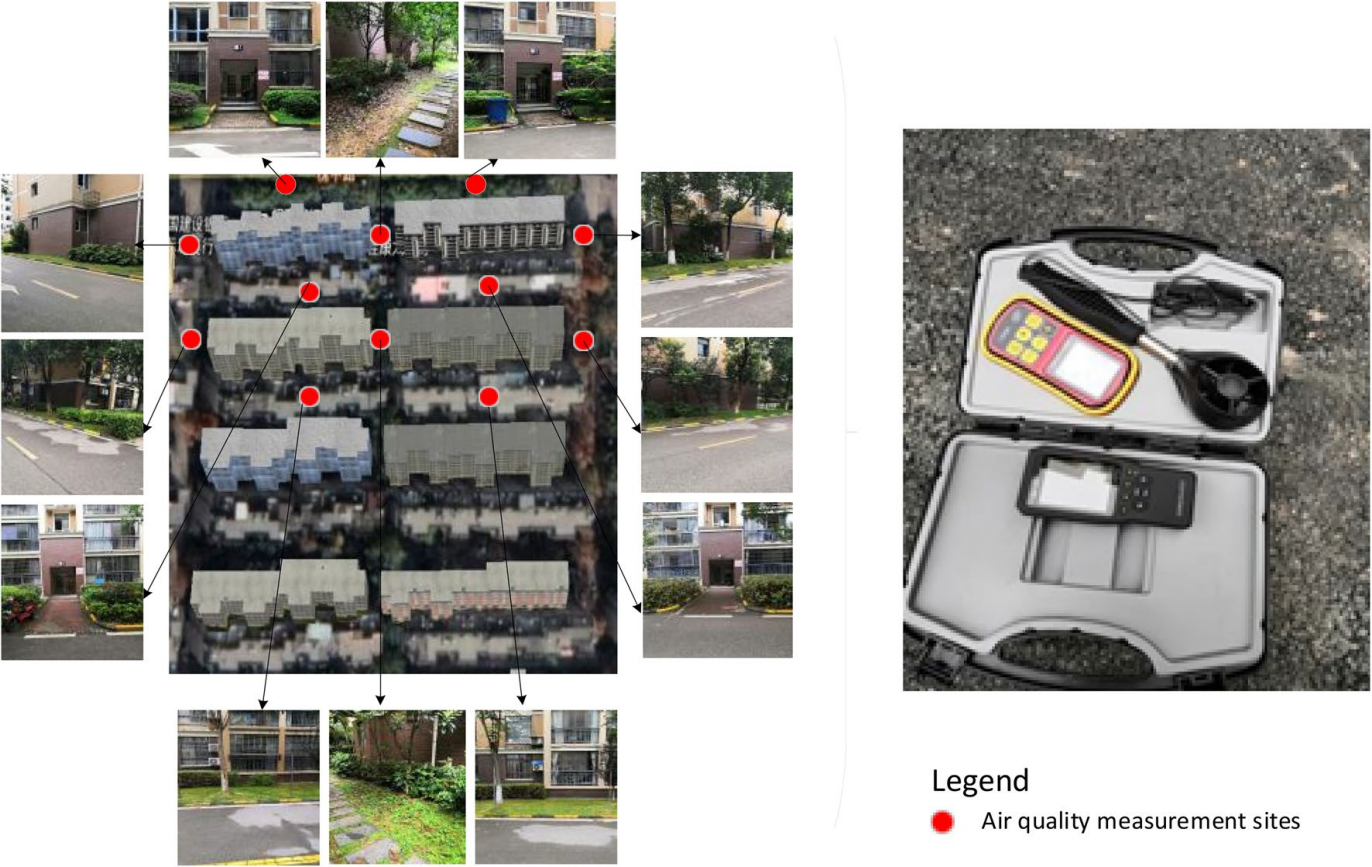
Distribution of field measurement sites. The aerial map was obtained via Baidu Map, and all the other images were taken by the authors.

Twelve measurement sites were selected in the community, and their locations are shown in Fig. 3. Field measurements of wind velocity and PM_2.5_ concentration at 1.5 m pedestrian height in these sites were done by using the anemometer (accuracy 0.1 m/s, range 0.7–30 m/s) and PM_2.5_ m (accuracy 0.001 μg/m^3^, range 0.012–9.999 μg/m^3^), respectively. The measurement locations were set up both in the windward and the leeward side of the building, as well as in the passageway between them. All locations could be considered as representative of the wind environment and PM_2.5_ pollution for the whole region. Data were collected on December 18th, 2019, between 6 and 7 pm because this is the time when most families are having outdoor activities after the dinner, and therefore exposed to the higher risk of air pollution. The wind velocity and PM_2.5_ concentration were measured every 20 min at each measurement location, and the average of three measurements within the hour was used as the final measured value.

### Numerical setup and calibration

#### Geometry and mesh

The numerical model was set up by using the standard *k*–*ε* model and the DPM method in Ansys Fluent to construct the building-tree layout. In the model configuration, the length, width and height of the buildings of parallel and staggered types were described as 56 m × 14 m × 50 m with the building interval as 26 m, while for the buildings of semi-enclosed and enclosure types, the dimensions are 112 m × 14 m × 50 m. These geometries were characterized based on the respective averages of the actual dimension of the buildings and their spacings in the examined samples. Greenery was modelled based on the typical greening plan within the research area as previously mentioned. Green areas were bounded by a circle of plants (height = 0.8 m), with some trees being much taller (approximated height = 6 m) in the center. The trunk was represented by a cylinder 2 m high and 0.5 m in diameter, while the crown was represented by a sphere 4 m in diameter.

**Figure 4:**
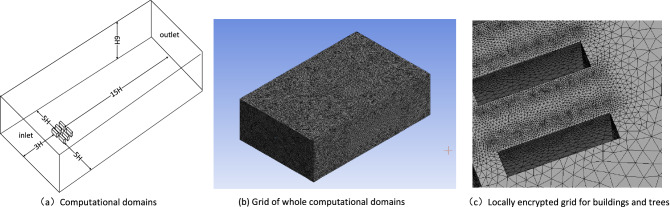
Computational domains and meshing.

It is also necessary to set up an enclosure around the community as the physical boundary. The size of the enclosure should be large enough to ensure that the simulation results are unaffected by the domain limitation. The current study constructed an enclosure of 10H × 18H × 6H as the computational domain, and H represented the height of the building (see Fig. 4). Subsequently, all elements in the computational domain were meshed, and the meshes of the trees and buildings were locally encrypted. The mesh number of the 11 geometric models varied between 4 and 9 million (see Fig. 4), and the mesh quality evaluation value all exceeded 0.8 (the evaluation value is usually between 0 and 1, the closer it is to 1, the better the mesh quality is), indicating a good quality of the constructed mesh.

#### Boundaries conditions

The steady solution was used to calculate the air flow field until the residual of each parameter decreased with the increase of the number of calculation steps, and finally tended to be flat to obtain the convergent calculation results. The top and both sides of the computational domain were set as symmetry, while the underside, building surfaces and tree trunks were all set as walls. The canopy was set as a porous medium because there were gaps in the canopy for air flows to pass. The setting of parameters mainly refers to the empirical values in the existing literature^25^, and the porosity, viscosity and inertia coefficients of the canopy were set as 0.4, 2.7e05 and 328.1, respectively. The Winter is the season in which air pollution is usually recorded to be the severest within the study area, and during this period people are often exposed to the highest risk linked to PM_2.5_ contact. As a result, when setting up the model, an average temperature of − 2.2 °C was adopted as recorded from 2013 to 2018, and an average wind speed of 2.5 m/s was selected, with the dominant wind direction chosen to be from the northeast or north.

The unsteady solution was used after the airflow simulation converged. The DPM model was employed to simulate the distribution of PM_2.5_ after it was injected into the computational domain from the bottom surface. PM_2.5_ emission sources around a community were treated as the non-point source pollution, which were input from the lower boundary of the computational domain. In the DPM model, the two-way coupling particle tracking method was used to allow for interactions between PM_2.5_ particles and the airflow. The time step was set to 1 s and the number of time steps was set to 500. The velocity magnitude of PM_2.5_ injection from the bottom surface was 1.5 m/s, and the total flow rate was 8.4e−09 kg/s. These values were set based on the real values collected within the field measurements. PM_2.5_ particles at the top and both sides of the computational domain were set to escape, those at the undersides, building surfaces and tree trunks were set to reflect, and those at canopies were set to trap. In 11 sets of models, all the numerical results converged within the set time step of 500, and the number of PM_2.5_ particles tracked varies from 200 to 400 thousand. The first time for particles to reach the tree canopy was less than 15 s for all the 11 models. The PM_2.5_ concentration in the air can be known according to the ‘DPM concentration’ derived from Ansys Fluent. PM_2.5_ emission sources around a community were treated as the non-point source pollution, which were input from the lower boundary of the computational domain.

#### Comparison of numerical simulation and on-site measurement

After collecting the field data, the calibration of numerical model was conducted, in which the numerical wind velocities and PM_2.5_ concentrations are compared with 12 measurement sites as shown in Fig. 3. The paired *t*-test was performed on two sets of data (i.e., measured and simulated) and the statistics obtained are shown in Table 2. It is shown that for both the wind velocity and PM_2.5_ concentration, the significance *p* (2-tailed) between the measured and simulated values exceeded 0.05, indicating that there is no significant difference between the physical observations and numerical simulations. Therefore, the proposed model is considered reliable in predicting the real design scenarios in the following sections.

**Table 2:** Comparisons of measured and numerical results in paired *t*-test.

	Mean	STD	*t*	*p*
Wind velocity	− 0.133	0.657	− 0.703	0.497
PM_2.5_ concentration	0.036	2.540	0.049	0.961

## Results

In this section the simulation results obtained of the wind velocity and PM_2.5_ concentration are presented. The following configurations as shown in Fig. 2 are investigated: (i) for Configuration-1 all the building-tree arrangements are in parallel; (ii) for Configuration-2, staggered; (iii) for Configuration-3 semi-enclosed; and (iv) for Configuration-4 enclosed.

### Configuration-1

**Figure 5:**
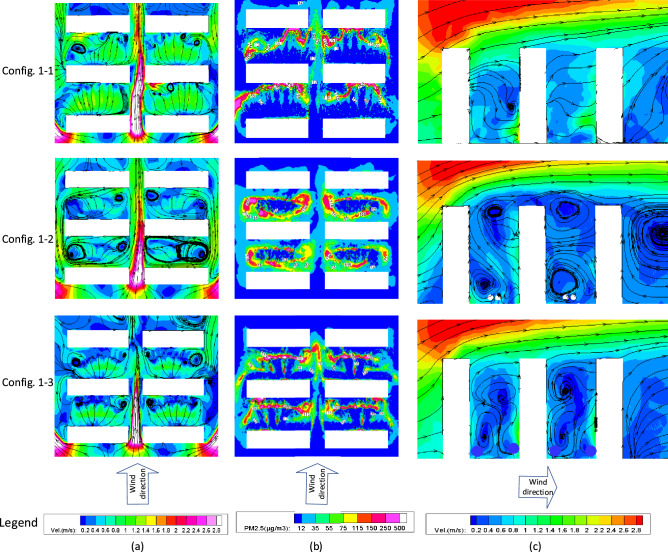
Plan view of the (**a**) wind velocity; (**b**) PM_2.5_ concentration at pedestrian height, and (**c**) lateral view of the wind velocity for Configuration-1.

Figure 5 shows the distributions of wind velocity and PM_2.5_ concentration at the pedestrian height (1.5 m above the ground) in a plan view, and the longitudinal profile of wind velocity in a lateral view. The three buildings are placed in parallel with an equal distance among them.

A narrow tube effect (the instantaneous increase in wind velocity caused by the close spacing between two buildings^26^) between the two columns of buildings can be observed in all three configurations in Fig. 5. These high winds enhance the dispersion of PM_2.5_, leading to a low particle concentration in the areas. However, it was commonly believed that wind comfort at pedestrian height can be divided as follows: 1 m/s ≤ wind velocity < 5 m/s, comfortable; 5 m/s ≤ wind velocity < 10 m/s, uncomfortable; 10 m/s ≤ wind velocity < 20 m/s, extremely uncomfortable; wind velocity > 20 m/s, dangerous^27^. An obvious increase in wind velocity between buildings may reduce the comfort for residents who use this passageway because the winter wind was usually intense. Also shown in Fig. 5, relatively low wind velocities are observed in the gaps between the adjacent buildings of each column, because the airflow has been effectively blocked by the surrounding buildings. The central shrubs have a more obvious effect on reducing the airflows than the trees because they are more closely planted, without which very large wind areas can be generated as shown in Figure 5 for Config. 1-1. Very few differences are observed in the wind velocity between the upstream and the downstream building gap spaces due to the symmetric configuration of buildings and trees. This also indicated an accurate numerical simulation and correct setting of boundary conditions.

Besides, Fig. 5 also shows that different tree-layouts lead to different distributions of PM_2.5_ in the space between the two buildings, because they are capable of changing the local wind velocity and direction, by serving as a barrier. The trees seem to have a more obvious influence on the wind field than the surrounding buildings. Low PM_2.5_ concentrations usually occur in the place where the airflow velocity is high, and this has been reflected within the numerical results. The aerodynamic effect of the trees on leeward side of the building is more evident in view of increasing airflow velocity and decreasing PM_2.5_ concentration at pedestrian height, as compared to the windward side of the building, due to the generation of air circulation in the former. It is important to mention that the trees near the windward side of a building tend to slightly reduce the wind speed, while the trees near the leeward side tend to increase it. Moreover, in Config. 1-2, the trees in the central space between the two buildings formed a shelter to the airflow and thus led to a circular area with high PM_2.5_ concentrations.

### Configuration-2

**Figure 6:**
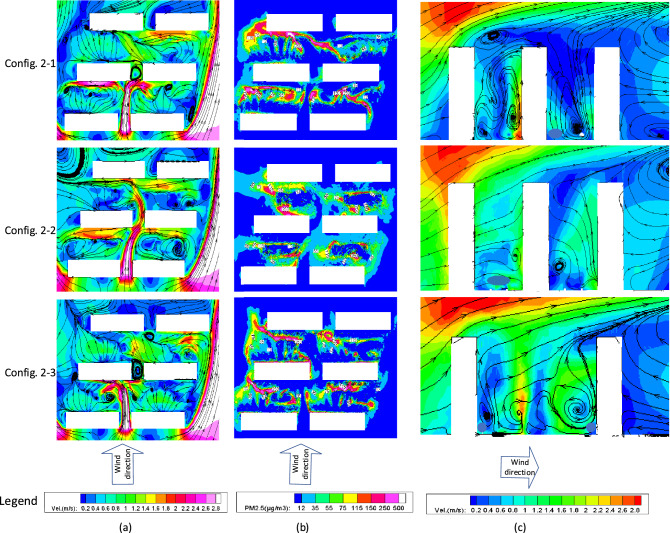
Plan view of the (**a**) wind velocity; (**b**) PM_2.5_ concentration at pedestrian height, and (**c**) lateral view of the wind velocity for Configuration-2.

Figure 6 shows the distributions of wind velocity and PM_2.5_ concentration at the pedestrian height, and the longitudinal profile of wind velocity, in the same way as Fig. 5. The three buildings are placed in stagger with an equal distance among them.

Figure 6 shows that the staggered type buildings developed a stronger wind area in the form of a narrow tube, due to the obstruction to the wind of the downstream building. The left and right-hand sides of the building arrays created the areas with low and high wind velocities, respectively. The former was mainly due to the fact that the wind hit the upstream face of the buildings and formed a large wind-shaded area behind, while the latter downstream protruding buildings did not benefit from this. Therefore, most areas on the left side of the buildings are characterized by relatively high concentrations of PM_2.5_ due to their slow dispersion process from the low wind. It should be noted that while the distribution of PM_2.5_ concentration in Config-1 is symmetrical, this behavior is not visible for Config-2 with a more irregular distribution of PM_2.5_ concentration.

According to the profiles shown in Fig. 6, the average wind velocity in the space between the staggered buildings is found to be slightly larger than that reported from the parallel type buildings in Fig. 5, because the dispersed airflow from the windward blocks can easily reach the gaps behind. Trees have also shown their great capacity to change the local wind velocity and direction. Figures 5 and 6 suggest that both parallel and staggered type buildings share the same features in that the trees near the windward side of a building tended to reduce the local wind velocity, while the trees near the leeward side seemed to increase this. Moreover, dense tree layouts inside the building intervals could be unfavorable to the ventilation and lead to the accumulation of pollutants.

### Configuration-3 and Configuration-4

**Figure 7:**
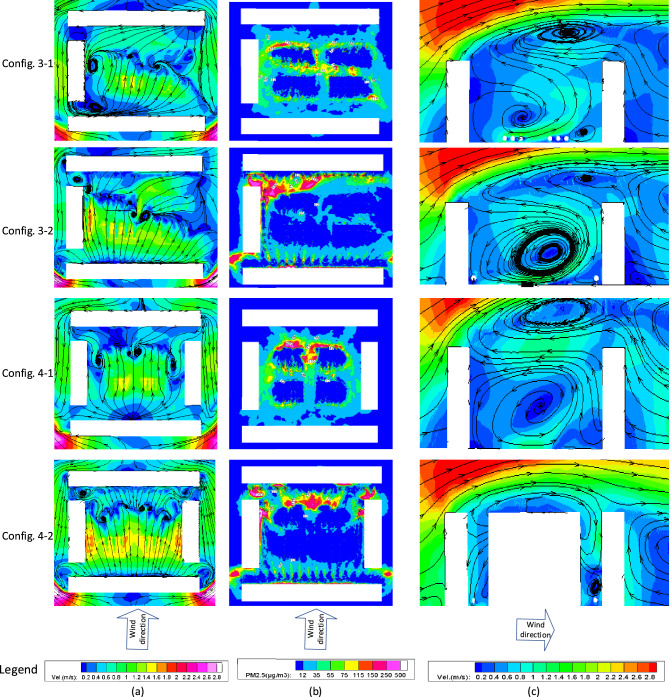
Plan view of the (**a**) wind velocity; (**b**) PM_2.5_ concentration at pedestrian height, and (**c**) lateral view of the wind velocity for Configuration-3 and -4.

Figure 7 shows the distributions of wind velocity and PM_2.5_ concentration at the pedestrian height in a plan view, and the longitudinal profile of wind velocity in a lateral view, for the semi-enclosure type (Config-3) and enclosure type buildings (Config-4).

In both cases, quite a few shadow areas with small wind velocity were formed on the downstream side of the building due to the block effect of air passageway by the front faces. Although the right-hand side of the semi-enclosed buildings Config-3 has no barrier, its opposite side was mostly blocked, making difficult for the airflow to reach the central space from the narrow passageway. As a result, in Config. 3-1 the average wind velocity on the open side of the building is higher than that on the closed side at the pedestrian height and, consequently, leading to a lower PM_2.5_ concentration due to the relatively rapid dispersion. On the other hand, the existence of trees around the buildings on the closed side in Config. 3-2 also increased the local wind velocity and reduced the PM_2.5_ concentrations. Furthermore, the airflow could not directly reach the very vicinity of the buildings in Config. 4-1 and 4-2 because of the blocking effect generated by the surrounding buildings, but the trees induced large wind field in the center. The distributions of wind velocity and PM_2.5_ concentration in Config-4 are nearly symmetric, indicating the correct simulation results in a qualitative way.

Generally, wider building spacings allow for more airflow access to the central space of the community, and alternatively the trees can change the local wind velocities. By comparing Config-3 and Config-4 in Fig. 7, it is notable that the combined building-tree layouts have a more substantial influence on the wind velocity than the design within which only one single factor is taken into consideration. Furthermore, the aerodynamic effects of the trees on the leeward side of the building seem to be more pronounced than those on the windward side. PM_2.5_ particles are mainly concentrated near the windward area of the downstream building and the dense tree area.

### Analysis of PM_2.5_ concentration within sample

According to *Ambient Air Quality Standards* (GB3095-2012) released by the Ministry of Ecology and Environment in China, PM_2.5_ pollution levels can be divided into the following categories based on their 24-h average concentration values (μg/m^3^): excellent (< 35), good (35–75), slight pollution (75–115), moderate pollution (115–150), heavy pollution (150–250) and serious pollution (more than 250). In practice, the 24-h average concentration of PM_2.5_ is required to be below < 75, above which it will cause adverse effect on people’s health. Table 3 shows the percentage of the areas within all the building-trees simulations for which the PM_2.5_ concentrations is above this threshold. Table 3 shows that the area ratio with PM_2.5_ concentrations exceeding 75 is higher for each community in Config-1 and 2, in comparison to Config-3 and 4.

**Table 3:** Percentage area with PM_2.5_ concentration over 75 μg/m^3^ in each configuration.

Building-tree arrangement	Percentage of area (%)
Parallel type buildings with trees around buildings (Config. 1-1)	13.33
Parallel type buildings with trees in central space (Config. 1-2)	14.35
Parallel type buildings with trees around buildings and in central space (Config. 1-3)	13.95
Staggered type buildings with trees around buildings (Config. 2-1)	12.25
Staggered type buildings with trees in central space (Config. 2-2)	11.43
Staggered type buildings with trees around buildings and in central space (Config. 2-3)	12.13
Semi-enclosed type buildings with trees in central space (Config. 3-1)	3.57
Semi-enclosed type buildings with trees around buildings and in central space (Config. 3-2)	9.71
Enclosure type buildings with trees in central space (Config. 4-1)	4.48
Enclosure type buildings with trees around buildings and in central space (Config. 4–2)	7.70

In principle the semi-enclosed or enclosed type buildings may lead to a higher PM_2.5_ concentration because they have more dimensions to block the wind, but the building spacing between these is relatively larger, which is more favorable to the air ventilation and the diffusion of pollutants^28^. The trees also have a critical influence on the airflow at the pedestrian height. The semi-enclosed type and enclosed type buildings are more suitable for the design of large green areas in their central spaces, and these plants could be capable of changing the wind speed and direction due to their resistance against the airflow. Comparatively, the green areas in the central space of parallel type and staggered type buildings are small, and this has resulted in a weaker aerodynamic effect.

On the other hand, the aerodynamic effect of the trees is not always evident in all the configurations within our simulation samples. The trees’ density, size, and position relative to the buildings all play a role. For example, in Config. 1-2, the trees in the narrow building spacing produced a shield to the airflows, leading to sharp increase of the pollutant concentration, so it has produced the largest area ratio with PM_2.5_ concentrations exceeding 75. In comparison, Config. 3-1 reported the lowest area ratio with PM_2.5_ concentration exceeding 75. Although the building on the left-hand side has been blocked from the airflow with a reduced wind velocity, it only caused a limited increase in PM_2.5_ concentration due to the large building intervals. In Config. 3-2, the effective use of trees around the buildings increased the vorticity, promoting the convective exchange of the airflow and thus the dispersion of suspended particles. However, the trees around the downstream buildings seemed to cause low turbulence, and therefore, they formed a sheltered zone to the airflow, resulting in a significant increase in PM_2.5_ concentrations. It seems that the inclusion of trees around the buildings in Config. 3-2, adversely, has increased the area with excessive pollution concentration. Therefore, an optimal building-tree layout plan including all possible influence factors should always be taken into consideration.

## Discussions

The above numerical results have revealed useful findings. Firstly, the aerodynamic effects of the trees on the windward side of the building were prone to decrease the wind velocity, hindering the PM_2.5_ diffusion. Contrarily, the trees on the leeward side of the building were prone to enhance the ventilation and thus, decrease the PM_2.5_ accumulation. This is in line with most studies on urban community scale which have emphasized the role of trees near buildings as the wind shields^29,30^, and other ones which have identified that planting trees is one of the effective ways to reduce areas with excessive wind speed in urban communities^23,31^. Secondly, most of these existing studies focused mainly on the influence of the type, porosity, quantity and configuration of trees on the wind environment^25,32^, neglecting the roles of the tree arrangement or building-tree relative position, as verified within this work. Trees in the wind shadow area on the leeward side of the buildings can also increase the turbulence and the wind velocity when the horizontal airflows by-passed the small shrubs. Therefore, the trees within urban communities may not always reduce the wind speed, but the effect is related to their positions.

Many previous studies on the street canyon scale have produced abundant conclusions by comparing the levels of pollutants with and without the trees in urban areas^15^. Some studies have emphasized the pollutant removal capacity from the presence of roadside vegetation^33,34^, while others suggested that the trees could reduce the ventilation and hider the dilution of the pollutants^35,36^. The reasons for these contradictory conclusions are associated with the fact that other elements such as the urban morphology, building geometry and climatic conditions, may not be consistent or identical within all these studies, and therefore they played an additional role on the wind environment and pollutants dispersion^37^. A similar situation also exists at the present urban community scale, where the building geometry, tree morphology and relative position of the buildings and trees, all have potential effects.

The previous studies reported that the parallel type and enclosure type buildings were less ventilated than the staggered type and semi-enclosed type buildings^26^. However, the current study found that for similar tree arrangements, the communities with parallel type and staggered type buildings have a larger area affected by PM_2.5_ concentration exceeding 75 μg/m^3^ when compared with the communities characterized by the semi-enclosed type and enclosed type buildings. This may be due to that the current study focused on the excessive PM_2.5_ concentrations, while ventilations could improve the dispersion of the particles of many pollutants and reduce their average concentrations in a community which may not necessarily be PM_2.5_.

The narrow space between the trees near the windward side of the rear building and the trees located within the building center could form a dense vegetation area, which increases the PM_2.5_ concentration due to their function of reducing the turbulence and hindering the ventilation. Previous studies on the urban scale or regional scale suggested that large areas of clustered trees can be conducive to the purification of air pollutions because this arrangement plays a significant role in the deposition of pollutants^37,38^. However, other articles have suggested that the aerodynamic effects of the trees are much more significant instead^36,39^, which lead to a similar conclusion with the results obtained in the present study. It is important to state the clustered tree arrangements in urban communities may form a wind shelter, a condition which is unfavorable to the ventilation and diffusion of pollutants.

Increasing the building intervals could improve the wind ventilation when the greening ratio and the position of trees relative to the buildings are kept constant. This is also in line with what have been found by others on the influence of buildings on the wind environment. Some of these have reported a linear and direct relationship between the building interval and the ventilation without considering the presence of trees^40,41^. A wide building interval can allow more airflows into the community, thus improving the ventilation condition.

These findings can provide a significant reference for improving the design of the built environment for future urban communities in view of reducing people’s exposure to PM_2.5_ and health risks. However, there also existed some limitations that need to be highlighted. For example, in addition to the aerodynamic effects on the pollutant dispersion, vegetations in the community can have dipositive and absorptive effects on the pollutants. Besides, the natural winds always have time-varying velocities^42^, while the current study simplified the wind characteristics as a constant value. In addition, many studies have proved that the wind direction has a significant impact on the air circulation between buildings^43–45^. Therefore, future research should focus on the effects of wind profile in the atmospheric boundary layer and different wind directions for prevailing wind condition on wind fields under different building-tree configurations.

## Conclusions

Buildings and trees are the two important elements in the urban environment. Their shape and layout can affect the wind condition and the dispersion of air-pollutants, thus playing a strong role on the health of urban residents. The evaluation of the influence of different urban environments on the outdoor pollutant dispersions can provide useful references for understanding the risk and optimizing the sustainable design of urban living environment. The current study extracted 10 typical building-tree arrangements from 120 communities in Shijiazhuang City, which is one of the most polluted cities in China. The aerodynamic effects of the buildings and trees on pollutant dispersion were numerically simulated, and the results revealed a close link between the building-tree arrangements and the air-pollutants dispersion.

The results have shown that: (1) a wide building interval space could benefit the air ventilation and thus decrease PM_2.5_ concentrations, however, this effectiveness is highly influenced by the presence of the trees; (2) the trees on the leeward side of a building tend to increase the local wind velocity and decrease the pedestrian-level PM_2.5_ concentrations, while those on the windward side tend to decrease the wind velocity. The small distance with trees in the central space of the community forms a wind shelter, hindering the particles' dispersion; and (3) the configuration of parallel type buildings with clustered tree layouts in the narrow central space is most unfavorable to the air ventilation, leading to larger areas affected by excessive PM_2.5_ concentration.

## Data Availability

The datasets used and/or analysed during the current study available from the corresponding author on reasonable request.
